# Participants' Evaluation of the Project P.A.T.H.S.: Are Findings Based on Different Datasets Consistent?

**DOI:** 10.1100/2012/187450

**Published:** 2012-06-04

**Authors:** Daniel T. L. Shek, Rachel C. F. Sun

**Affiliations:** ^1^Department of Applied Social Sciences, The Hong Kong Polytechnic University, Hong Kong; ^2^Public Policy Research Institute, The Hong Kong Polytechnic University, Hong Kong; ^3^Department of Social Work, East China Normal University, Shanghai 200241, China; ^4^Kiang Wu Nursing College of Macau, Macau; ^5^Division of Adolescent Medicine, Department of Pediatrics, Kentucky Children's Hospital, University of Kentucky College of Medicine, Lexington, KY 40506-9983, USA; ^6^Faculty of Education, The University of Hong Kong, Hong Kong

## Abstract

Subjective outcome evaluation findings based on the perspective of the participants of the Project P.A.T.H.S. (Positive Adolescent Training through Holistic Social Programmes) in nine datasets collected from 2005 to 2009 (*n* = 206, 313 program participants) were examined in this paper. Based on the consolidated data with schools as units, results showed that the participants generally had positive perceptions of the program, implementers, and benefits of the program. More than four-fifths of the participants regarded the program as beneficial to their holistic development. Multiple regression analysis revealed that the perceived qualities of the program and the program implementers predicted perceived effectiveness of the program. Based on the subjective outcome evaluation findings, the present study provides support for the effectiveness of the Tier 1 Program of the Project P.A.T.H.S. in Hong Kong.

## 1. Introduction

In his review of adolescent developmental issues in Hong Kong, Shek [1] drew several conclusions based on the available statistics and research findings. First, the adolescent substance abuse problem was part of a changing scene in Hong Kong. Second, although the overall youth crime trend was relatively stable in the past decade, the crimes of shoplifting and stealing deserved our concern. Third, the adolescent mental health problem was a growing issue. Fourth, adolescent unhealthy life styles, such as smoking, early sex, and moral confusion, were issues of concern. Fifth, adolescents experiencing economic disadvantage were a growing problem in Hong Kong, with one-quarter of children and adolescents experiencing economic disadvantage. Sixth, unemployed and nonengaged youth were emerging problems in the past 2 decades. Seventh, family and parenting problems in families with adolescents deserved our attention, and the Social Development Index showed that there was a gradual drop in family solidarity in the past decade. In the past few years, adolescent substance abuse has appeared to deteriorate, and it is a topic attracting much public attention in Hong Kong [2, 3]. With reference to these adolescent developmental problems, one important question is how we can promote holistic development in young people in Hong Kong.

To promote holistic development among adolescents in Hong Kong, The Hong Kong Jockey Club Charities Trust approved HK$400 million to launch a project entitled “P.A.T.H.S. to Adulthood: A Jockey Club Youth Enhancement Scheme” based on the perspective of positive youth development. The word “P.A.T.H.S.” denotes Positive Adolescent Training through Holistic Social Programmes. There are two tiers of programs (Tier 1 and Tier 2 Programs) in the P.A.T.H.S. Project. Whereas the Tier 2 Program is a selective program for students with greater psychosocial needs, the Tier 1 Program is a universal positive youth development program in which students in secondary 1 to 3 participate, normally, with 20 h of training in the school year at each grade, involving 40 teaching units that have been developed with reference to 15 positive youth development constructs [4]. These constructs include promotion of bonding, cultivation of resilience, promotion of social competence, promotion of emotional competence, promotion of cognitive competence, promotion of behavioral competence, promotion of moral competence, cultivation of self-determination, promotion of spirituality, development of self-efficacy, development of a clear and positive identity, promotion of beliefs in the future, provision of recognition for positive behavior, provision of opportunities for prosocial involvement, and fostering prosocial norms. Because of the overwhelming success of the program, the project has been extended for another cycle, from 2009 to 2012, with an additional earmarked grant of HK$350 million.

There were two implementation phases in the original phase of the project—the experimental implementation phase and the full implementation phase. For the experimental implementation phase (January 2006 to August 2008), 52 secondary schools participated in the project with the objectives of accumulating experience in program implementation and familiarizing the front-line workers with the program design and philosophy. In the 2006/2007 school year, the programs were implemented on a full scale at the secondary 1 level. In the 2007/2008 school year, the programs were implemented at the secondary 1 and 2 levels. In the 2008/2009 school year, the programs were implemented at the secondary 1, 2, and 3 levels. The experimental and full implementation phases for the first cycle were successfully completed [5].

To provide a comprehensive picture pertaining to the effectiveness of the project, a wide range of evaluation strategies were employed to examine the program effect, including objective outcome evaluation utilizing a randomized group trial; subjective outcome evaluation based on quantitative and qualitative data collected from the program participants and instructors; qualitative evaluation based on focus groups involving students and instructors; in-depth interviews with program implementers; student products, such as weekly diaries; process evaluation involving systematic observations of delivery of the program and interim evaluation. The available evaluation findings consistently provide strong evidence that the Project P.A.T.H.S. has a beneficial influence on students [6–9].

To examine the perceptions of the program participants concerning the effectiveness of the project, subjective outcome evaluation or the client satisfaction approach was used. In human services, the importance of involving service users or program participants in evaluation is advocated, and thus subjective outcome evaluation becomes popularly used to capture the viewpoints of the participants. To capture the viewpoint of the participants, client satisfaction surveys are commonly used as feedback for transforming services, to meet the users' needs for planning and administration purposes, or simply as an indicator of program effectiveness from the participants' perspective for research purposes. Although there are many criticisms of this approach, the client satisfaction approach is widely used in different service settings. As pointed out by Royse [10], “despite the generally positive bias and the problems associated with collecting representative samples of clients, there is much to recommend client satisfaction studies as one means of evaluating a program. Because professionals do not experience the agency in the same way as the clients, it is important to ask clients to share their experiences” (pp. 264-265).

Subjective outcome evaluation is a popular approach employed by different professionals in different fields, such as education, social work, psychology, medicine, and allied health professions. The commonly used method develops closed-ended rating scale items to quantify client satisfaction. For example, standardized rating scales, such as the medical interview satisfaction scale, consumer satisfaction questionnaire, and client satisfaction questionnaire, were developed to gauge client satisfaction and perceived helpfulness of the program. In fact, it is commonly argued that, with the use of valid and reliable measures of the perceptions of the program participants, subjective outcome evaluation can yield objective pictures about program evaluation.

Previous studies showed that roughly four-fifths of the program participants generally had positive perceptions of the program, instructors, and benefits of the P.A.T.H.S. Project. In addition, the findings are fairly stable in different cohorts of students in the experimental and full implementation phases [11–13]. Furthermore, program content and program instructors were found to be significant predictors of perceived benefits of the program. As the Project P.A.T.H.S. was implemented in different cohorts of students from 2005 to 2009, it would be illuminating to aggregate findings in different cohorts to form an overall picture regarding the satisfaction of the participants. With data collected from large samples over time, a more stable picture of the subjective outcome evaluation findings can be generated. Against this background, the present paper attempts to describe the profile of subjective outcome evaluation findings based on the perspective of the participants. In addition, predictors of the perceived effectiveness of the program were also examined in this study involving aggregation of different datasets.

## 2. Methods

### 2.1. Participants and Procedures

From 2005 to 2009, the total number of schools that participated in the Project P.A.T.H.S. was 244, with 669 schools in the secondary 1 level, 443 in the secondary 2 level, and 215 in the secondary 3 level (Table 1). Altogether, 223,101 students participated in the Tier 1 Program in these 5 years. In these three grades, the mean number of students per school was 167.28 (range: 5–280), with an average of 4.61 classes per school (range: 1–8). Among them, 46.27% of the respondent schools adopted the full program (i.e., 20 h program involving 40 units), whereas 53.73% of the respondent schools adopted the core program (i.e., 10 h program involving 20 units). The mean number of sessions used to implement the program was 22.77 (range: 3–66). While 51.54% of the respondent schools incorporated the program into the formal curriculum (e.g., liberal studies, life education), 48.46% used other modes (e.g., form teachers' periods and other combinations) to implement the program.

After completing the Tier 1 Program, the students were invited to respond to a Subjective Outcome Evaluation Form for Students (Form A) developed by the first author. From 2005 to 2009, a total of 206,313 questionnaires were completed (104,717 for the secondary 1 level, 69,334 for the secondary 2 level, and 32,262 for the secondary 3 level). The overall response rate was 92.48%. To facilitate the program evaluation, the research team developed an evaluation manual with standardized instructions for collecting the subjective outcome evaluation data. In addition, adequate training was provided to the implementers during the 20 h training workshops on how to collect and analyze the data collected by Form A.

On the day when the evaluation data were collected, the purpose of the evaluation was mentioned and the confidentiality of the data collected was repeatedly emphasized to all of the respondents. The respondents were asked to indicate if they did not want to respond to the evaluation questionnaire (i.e., “passive” informed consent was obtained). All respondents responded to all scales in the evaluation form in a self-administration format. Adequate time was provided for the respondents to complete the questionnaire.

### 2.2. Instruments

The Subjective Outcome Evaluation Form (Form A) [11–13] was used to measure the program participants' perceptions of the Tier 1 Program. Broadly speaking, there are several parts in this evaluation form as follows:

- participants' perceptions of the program, such as program objectives, design, classroom atmosphere, interaction among the students, and the respondents' participation during class (10 items)
- participants' perceptions of the workers, such as the preparation of the instructor, professional attitude, involvement, and interaction with the students (10 items)
- participants' perceptions of the effectiveness of the program, such as promotion of different psychosocial competencies, resilience, and overall personal development (16 items)
- the extent to which the participants would recommend the program to other people with similar needs (1 item)
- the extent to which the participants would join similar programs in the future (1 item)
- overall satisfaction with the program (1 item)
- things that the participants learned from the program (open-ended question)
- things that the participants appreciated most (open-ended question)
- opinion about the instructor(s) (open-ended question)
- areas that require improvement (open-ended question).

For the quantitative data, the implementers collecting the data were requested to input the data into an EXCEL file developed by the research team that would automatically compute the frequencies and percentages associated with the different ratings for an item. When the schools submitted the reports, they were also requested to submit the soft copy of the consolidated datasheets. After receiving the consolidated data by the funding body, the data were aggregated in order to “reconstruct” the overall profile based on the subjective outcome evaluation data by the research team.

### 2.3. Data Analyses

Percentage findings were examined using descriptive statistics. A composite measure of each domain (i.e., perceived qualities of program content, perceived qualities of program implementers, and perceived program effectiveness) was created based on the total scores of each domain divided by the number of items. Pearson's correlation analysis was used to examine if the program content and program implementers were related to the program effectiveness. Multiple regression analysis was performed to compare which factor would predict the program effectiveness. All analyses were performed by using the Statistical Package for Social Sciences version 17.0.

## 3. Results

The quantitative findings based on the closed-ended questions are presented in this paper. Several observations can be highlighted from the findings. First, the participants generally had positive perceptions of the program (Table 2), including clear objectives of the curriculum (83.50%), much interaction among students (81.84%), and well-planned teaching activities (81.76%). Second, a high proportion of the participants had a positive evaluation of implementers' performance (Table 3). For example, the participants thought that the implementers were very involved (88.63%), ready to help them when needed (88.22%), and encouraged them to participate in activities (88.12%). Third, as shown in Table 4, many participants perceived that the program promoted their development, including moral competence (84.05%), compassion for others (81.75%), social competence (82.11%), and overall development (83.24%). Fourth, 77% of the participants would recommend the program to students with similar needs. Fifth, 65.37% of the participants expressed that they would participate in similar courses again in the future. Finally, 84.48% of the respondents were satisfied with the program on the whole (Table 5).

Reliability analysis with the schools as the unit of analyses showed that Form A was internally consistent (Table 6): 10 items related to the program (*α* = 0.98), 10 items related to the implementer (*α* = 0.99), 16 items related to the benefits (*α* = 1.00), and 36 items measuring overall program effectiveness (*α* = 0.99). Results of correlation analyses showed that both program content (*r* = 0.85, *P* < 0.01) and program implementers (*r* = 0.74, *P* < 0.01) were strongly associated with program effectiveness (Table 7). 

Table 8 presents multiple regression analysis results. Higher positive views toward the program and program implementers were associated with higher program effectiveness (*P* < 0.01). Further analyses showed that program content (*β* = 0.75) was a significantly stronger predictor than program implementers (*β* = 0.24). This model explained 95% of the variance toward the prediction of program effectiveness. Interestingly, the above relationships and the amount of variance were consistent across grade levels.

## 4. Discussion

The purpose of this study was to evaluate the Tier 1 Program of the Project P.A.T.H.S. via the subjective outcome evaluation approach based on the perspective of the program participants using the data collected in the experimental and full implementation phases (2005–2009) of the project. There are several characteristics of this study. First, a large sample of schools (more than 200 schools per grade) and students (*n* = 206, 313) were involved. Second, different datasets collected at different points of time were analyzed in this study. Third, responses of students in different grades were collected. Fourth, this is the first known scientific study of the subjective outcome evaluation of a positive youth development program based on different cohorts in China. Finally, this is also the first study of subjective outcome evaluation based on such a large sample of participants in the global context.

Generally speaking, the quantitative findings showed that a high proportion of the respondents had positive perceptions of the program and the workers; roughly four-fifths of the respondents regarded the program as helpful to them. The findings basically replicated those findings reported previously based on the perspective of the program participants and they are also consistent with those based on the perspective of the program implementers. In fact, an examination of the percentages of responses to different items revealed that the figures were very similar across different studies. In conjunction with findings based on other evaluation strategies, the present integrative evaluation study showed that the Tier 1 Program of the Project P.A.T.H.S. was well received by the program participants, and over four-fifths of them were of the view that the program was beneficial to their development.

There are several contributions of the present study. First, in view of the lack of positive youth development programs and related evaluation findings in the Chinese context, the present study is a pioneer study. Besides showing that Project P.A.T.H.S. is effective, it also demonstrates how subjective outcome evaluation based on a large sample size can be carried out. Second, the findings show that the subjective outcome measure is reliable. Because there are few validated measures in the Chinese culture [14, 15], the present study contributes to the assessment literature on psychosocial measures in the Chinese context.

Finally, findings on the predictors of subjective outcome evaluation are important because there are currently few conceptual models on the determinants of subjective outcomes. There has been some discussion in the literature on how the quality of a program can be enhanced by tailoring an appropriate program to suit the values and needs of target populations [16, 17]. For example, using the Youth Program Quality Assessment (YPQA) instrument, researchers found that the effect of program delivery qualities varied with the students' ages [18]. In addition, the positive youth-oriented approach was found to be more beneficial for high-school students, while staff-oriented pedagogy was more appropriate for elementary school students [17, 18]. Unfortunately, although program components and their interactions with individual factors are important determinants of the effectiveness of youth programs, very few studies have examined the effect of different program components on perceived program effectiveness, especially in the Chinese context. The present findings fill an important gap in the formulation of theoretical models on the determinants of subjective outcome evaluation of positive youth development programs.

Although utilization of subjective outcome evaluation or the client satisfaction approach in evaluation has a long history in human services, there are arguments against the use of subjective outcome evaluation. For example, subjective outcome evaluation has been criticized as biased and unable to reflect the real behavioral changes in the program participants [19, 20]. Nevertheless, there are several features in this study that may be used to argue against such criticisms. First, a very big sample was used in this study, with 206,313 students in roughly half of the secondary schools in Hong Kong. Such a big sample size substantially enhances the generalizability of the research findings and their credibility. Second, different aspects of subjective outcome, including views on the program, worker, perceived effectiveness, and overall satisfaction, were covered in the study. The present findings also showed that the Form A rating items were reliable with reference to the sections and the whole scale. According to Royse [10], the lack of standardized assessment tools for conducting a client satisfaction survey also introduces biases for the client satisfaction approach. As such, he recommended the use of an assessment tool with known reliability and validity that would “eliminate many of the problems found in hastily designed questionnaires” (p. 265). 

Third, because the findings reported in this paper were “reconstructed” based on the reports submitted anonymously by the participating schools, the possibility that the students reported in an over-cooperative manner was not high. Finally, previous research findings based on the same project have shown that subjective outcome evaluation findings actually converged with objective outcome evaluation findings [21, 22]. In view of the lack of research data in this view, such studies point to the value of collecting subjective outcome evaluation data. Of course, the use of schools as the units of analyses might mask individual differences involved. However, in view of the large number of schools involved, this is not a particularly acute problem.

Despite these limitations, the present findings suggest that the Tier 1 Program of the Project P.A.T.H.S. and its implementation were perceived in a positive manner by the program participants. In conjunction with other evaluation findings, the present study suggests that the Tier 1 Program of the Project P.A.T.H.S. was perceived to be beneficial to the development of the program participants. With reference to the gradual decline of parental control in the early teenage years of Chinese adolescents in Hong Kong, positive youth development programs such as the Project P.A.T.H.S. are important initiatives to promote psychosocial competencies in Chinese adolescents of Hong Kong [23]. Furthermore, although subjective outcome evaluation is a popular approach used in human services in the Western contexts [24–28], there are comparatively few published studies in the Chinese contexts, particularly in the area of positive youth development. As such, the present integrative study and the related studies can be regarded as groundbreaking in the field of positive youth development in different Chinese contexts.

## Figures and Tables

**Table 1:** Description of data characteristics from 2005 to 2009.

	S1				S2			S3	
	2005/06 EIP	2006/07 FIP	2007/08 FIP	2008/09 FIP	2006/07 EIP	2007/08 FIP	2008/09 FIP	2007/08 EIP	2008/09 FIP
Total schools that joined P.A.T.H.S.	52	207	213	197	49	196	198	48	167
(i) 10 h program	23	95	108	104	27	113	110	29	104
(ii) 20 h program	29	112	105	93	22	83	88	19	63

Tier 1 Program:									
Mean no. of sessions of program implementation	17.75 (3–50)	23.55 (2–50)	23.61 (5–60)	23.54 (5–65)	23.76 (10–40)	22.81 (7–60)	23.04 (4–48)	24.07 (10–44)	22.78 (7–66)
No. of schools incorporated into formal curriculum	21	101	116	98	26	108	99	30	85
No. of schools incorporated into other modes	31	106	97	99	23	88	99	18	82
Mean no. of classes per school	4.58 (2–7)	4.66 (1–8)	4.69 (1–8)	4.56 (1–8)	4.51 (1–7)	4.62 (1–8)	4.64 (1–8)	4.56 (1–8)	4.67 (1–8)
Total no. of students	8679	35,735	36,343	31,280	8167	33,449	33,583	7708	28,157
Mean no. of students per school	166.90 (37–240)	172.63 (17–280)	171.05 (16–267)	158.78 (5–251)	166.67 (32–240)	170.66 (12–280)	169.61 (15–263)	160.58 (26–240)	168.60 (28–240)
Total no. of student respondents	8,057	33,693	33,867	29,100	7,406	30,731	31,197	6,830	25,432
Mean no. of student respondents per school	154.94 (37–212)	162.77 (15–265)	159.00 (14–267)	147.72 (3–251)	151.14 (32–220)	156.80 (12–243)	157.56 (15–263)	142.29 (23–213)	152.29 (22–229)

*Note:* S1: secondary 1 level; S2: secondary 2 level; S3: secondary 3 level; EIP: Experimental Implementation Phase, FIP: Full Implementation Phase.

**Table 2:** Summary of the students' perception towards the program.

		Respondents with positive responses (options 4–6)							
		S1		S2		S3		Overall	
		*n*	%	*n*	%	*n*	%	*n*	%
(1)	The objectives of the curriculum were very clear.	87,337	83.96	56,778	82.43	26,979	84.11	171,094	83.50
(2)	The design of the curriculum was very good.	83,446	80.30	53,948	78.41	25,821	80.55	163,215	79.75
(3)	The activities were carefully planned.	84,793	81.75	55,532	80.83	26,465	82.70	166,790	81.76
(4)	The classroom atmosphere was very pleasant.	81,986	79.18	54,047	78.79	26,137	81.76	162,170	79.91
(5)	There was much peer interaction among the students.	83,730	81.21	55,507	81.16	26,486	83.15	165,723	81.84
(6)	Students participated actively during lessons (including discussions, sharing, games, etc.).	84,124	81.08	54,932	79.97	25,896	80.91	164,952	80.65
(7)	The program had a strong and sound theoretical support.	79,513	76.69	52,063	75.78	25,018	78.17	156,594	76.88
(8)	The teaching experience I encountered enhanced my interest in the course.	79,692	77.11	51,635	75.35	24,872	77.88	156,199	76.78
(9)	Overall speaking, I have a very positive evaluation of the program.	78,676	75.96	51,580	75.13	25,049	78.33	155,305	76.47
(10)	On the whole, I like this curriculum very much.	79,811	77.27	51,527	75.19	24,944	78.13	156,282	76.86

*Note: *all items are on a 6-point Likert scale with 1 = strongly disagree, 2 = disagree, 3 = slightly disagree, 4 = slightly agree, 5 = agree, 6 = strongly agree. Only respondents with positive responses (options 4–6) are shown in the table.

**Table 3:** Summary of the students' perception towards the performance of program implementers.

		Respondents with positive responses (options 4–6)							
		S1		S2		S3		Overall	
		*n*	%	*n*	%	*n*	%	*n*	%
(1)	The instructor(s) had a good mastery of the curriculum.	89,359	86.21	58,707	85.52	28,035	87.49	176,101	86.41
(2)	The instructor(s) was well prepared for the lessons.	91,324	88.18	59,819	87.19	28,313	88.36	179,456	87.91
(3)	The instructor(s)' teaching skills were good.	89,201	86.33	57,929	84.64	27,734	86.66	174,864	85.88
(4)	The instructor(s) showed good professional attitudes.	90,771	87.79	59,356	86.63	28,179	87.99	178,306	87.47
(5)	The instructor(s) was very involved.	91,902	88.85	60,149	87.80	28,558	89.25	180,609	88.63
(6)	The instructor(s) encouraged students to participate in the activities.	91,453	88.49	59,791	87.26	28,350	88.60	179,594	88.12
(7)	The instructor(s) cared for the students.	89,526	86.59	58,496	85.34	27,864	87.08	175,886	86.34
(8)	The instructor(s) was ready to offer help to students when needed.	91,220	88.25	59,903	87.47	28,467	88.93	179,590	88.22
(9)	The instructor(s) had much interaction with the students.	87,310	84.41	57,329	83.64	27,562	86.07	172,201	84.71
(10)	Overall speaking, I have very positive evaluation of the instructors.	91,458	88.24	59,992	87.43	28,511	88.99	179,961	88.22

*Note: *all items are on a 6-point Likert scale with 1 = strongly disagree, 2 = disagree, 3 = slightly disagree, 4 = slightly agree, 5 = agree, 6 = strongly agree. Only respondents with positive responses (options 4–6) are shown in the table.

**Table 4:** Summary of the students' perception towards the program effectiveness.

		Respondents with Positive Responses (Options 3–5)							
		S1		S2		S3		Overall	
		*n*	%	*n*	%	*n*	%	*n*	%
	The extent to which the course (i.e., the program that all students have joined) has helped you								
(1)	It has strengthened my bonding with teachers, classmates, and my family.	80,951	77.97	52,227	76.04	25,008	78.28	158,186	77.43
(2)	It has strengthened my resilience in adverse conditions.	83,598	80.59	53,837	78.43	25,707	80.53	163,142	79.85
(3)	It has enhanced my social competence.	85,847	82.89	55,517	81.02	26,272	82.43	167,636	82.11
(4)	It has improved my ability in handling and expressing my emotions.	85,024	82.11	54,974	80.24	26,026	81.69	166,024	81.35
(5)	It has enhanced my cognitive competence.	84,679	81.80	54,765	79.93	25,952	81.41	165,396	81.05
(6)	My ability to resist harmful influences has been improved.	86,182	83.30	55,872	81.52	26,387	82.75	168,441	82.52
(7)	It has strengthened my ability to distinguish between the good and the bad.	87,909	84.94	56,851	83.02	26,809	84.18	171,569	84.05
(8)	It has increased my competence in making sensible and wise choices.	86,504	83.61	56,168	82.02	26,444	83.02	169,116	82.88
(9)	It has helped me to have life reflections.	83,686	80.84	54,753	79.94	26,111	81.96	164,550	80.91
(10)	It has reinforced my self-confidence.	82,632	79.88	53,058	77.49	25,093	78.77	160,783	78.71
(11)	It has increased my self-awareness.	84,337	81.54	54,135	79.03	25,813	80.99	164,285	80.52
(12)	It has helped me to face the future with a positive attitude.	84,703	81.92	54,804	80.06	26,135	82.02	165,642	81.33
(13)	It has helped me to cultivate compassion and care about others.	84,892	82.06	55,279	80.73	26,252	82.45	166,423	81.75
(14)	It has encouraged me to care about the community.	82,269	79.58	53,431	78.02	25,276	79.73	160,976	79.11
(15)	It has promoted my sense of responsibility in serving the society.	83,747	80.93	54,230	79.15	25,580	80.57	163,557	80.22
(16)	It has enriched my overall development.	86,743	83.80	56,245	82.12	26,596	83.81	169,584	83.24

*Note: *all items are on a 5-point Likert scale with 1 = unhelpful, 2 = not very helpful, 3 = slightly helpful, 4 = helpful, 5 = very helpful. Only respondents with positive responses (options 3–5) are shown in the table.

**Table:** (a) If your friends have needs and conditions similar to yours, will you suggest him/her to join this course?

Respondents with positive responses (Options 3-4)							
S1		S2		S3		Overall	
*n*	%	*n*	%	*n*	%	*n*	%
82,177	79.86	51,261	75.20	24,078	75.94	157,516	77.00

*Note: *The item is on a 4-point Likert scale with 1 = definitely will not suggest, 2 = will not suggest, 3 = will suggest, 4 = definitely will suggest. Only respondents with positive responses (options 3-4) are shown in the table.

**Table:** (b) Will you participate in similar courses again in the future?

Respondents with positive responses (options 3-4)							
S1		S2		S3		Overall	
*n*	%	*n*	%	*n*	%	*n*	%
70,007	68.05	43,382	63.70	20,392	64.35	133,781	65.37

*Note: *The item is on a 4-point Likert scale with 1 = definitely will not participate, 2 = will not participate, 3 = will participate, 4 = definitely will participate. Only respondents with positive responses (options 3-4) are shown in the table.

**Table:** (c) On the whole, are you satisfied with this course?

Respondents with positive responses (options 4–6)							
S1		S2		S3		Overall	
*n*	%	*n*	%	*n*	%	*n*	%
87,596	85.19	56,692	83.21	26,975	85.04	171,263	84.48

*Note: *all items are on a 6-point Likert scale with 1 = very dissatisfied, 2 = moderately dissatisfied, 3 = slightly dissatisfied, 4 = slightly satisfied, 5 = moderately satisfied, 6 = very satisfied. Only respondents with positive responses (options 4–6) are shown in the table.

**Table 6:** Means, standard deviations, Cronbach's alphas, and mean of interitem correlations among the variables by grade.

	S1		S2		S3		Overall	
	*M*(SD)	*α* (mean^#^)	*M*(SD)	*α* (mean^#^)	*M*(SD)	*α* (mean^#^)	*M*(SD)	*α* (mean^#^)
Program content (10 items)	4.28 (0.29)	0.98 (0.85)	4.22 (0.32)	0.99 (0.89)	4.26 (0.31)	0.99 (0.87)	4.26 (0.31)	0.98 (0.87)
Program implementers (10 items)	4.62 (0.30)	0.99 (0.93)	4.54 (0.31)	1.00 (0.95)	4.58 (0.32)	1.00 (0.95)	4.59 (0.31)	0.99 (0.94)
Program effectiveness (16 items)	3.41 (0.26)	1.00 (0.94)	3.31 (0.28)	1.00 (0.95)	3.33 (0.29)	1.00 (0.95)	3.36 (0.28)	1.00 (0.94)
Total effectiveness (36 items)	3.99 (0.26)	0.99 (0.80)	3.91 (0.28)	0.99 (0.83)	3.94 (0.28)	0.99 (0.82)	3.95 (0.28)	0.99 (0.82)

^#^ Mean interitem correlations.

**Table 7:** Correlation coefficients among the variables.

Variable		1	2	3
(1)	Program content (10 items)	—		
(2)	Program implementers (10 items)	0.91**	—	
(3)	Program effectiveness (16 items)	0.85**	0.74**	—

**P < 0.01.

**Table 8:** Multiple regression analyses predicting program effectiveness.

	Predictors			
	Program content	Program implementers	Model	
	*β* ^ a^	*β* ^ a^	*R*	*R^2^*
S1	0.75**	0.24**	0.97	0.94
S2	0.78**	0.21**	0.98	0.95
S3	0.80**	0.18**	0.97	0.94
Overall	0.75**	0.24**	0.97	0.95

**^a^**Standardized coefficients.

**P < 0.01.
